# Cleft Palate, Interdisciplinary Diagnosis, and Treatment

**DOI:** 10.1155/2015/701850

**Published:** 2015-07-28

**Authors:** Pablo Antonio Ysunza, Maria Carmen Pamplona, Gabriela Repetto

**Affiliations:** ^1^Speech Services, Ian Jackson Craniofacial and Cleft Palate Clinic, Neuroscience Program, Beaumont Health, 3535 13 Mile Road, Royal Oak, MI 48073, USA; ^2^Cleft Palate Clinic, Hospital Gea Gonzalez, Calzada de Tlalpan 4800, 14000 Mexico City, DF, Mexico; ^3^Hablarte e Integrarte, AC, Mimosa 33, 03300 Mexico City, DF, Mexico; ^4^Center for Genetics and Genomics, Facultad de Medicina, Clinica Alemana, Universidad del Desarrollo, Avenida Las Condes 12438, 7710162 Santiago, Chile; ^5^Hospital Padre Hurtado, Avenida Esperanza 2150, San Ramon, 8880465 Santiago, Chile

Cleft lip and palate is the 4th most common congenital malformation and the 1st most common craniofacial anomaly. The incidence of cleft lip and palate varies from 1 per 750 live births to 1 per 650 live births depending on the geographical area. Cleft palate is a feature of over 200 well defined syndromes of congenital malformations.

Although at the present time it is still not feasible to completely prevent the occurrence of cleft palate, the consequences of this major malformation on maxillary structure, esthetic appearance, speech, feeding, and swallowing can be appropriately addressed and corrected by the intervention of an interdisciplinary team.

A palatal cleft creates feeding difficulties and affects the function of the velopharyngeal sphincter during speech. The velopharyngeal sphincter is one of the most important valves of the vocal tract. Its function during speech is to keep a balanced nasal resonance and sufficient intraoral pressure by sealing and opening the communication between the nasal cavities and the rest of the vocal tract.

The velopharyngeal sphincter has 3 main components: the velum or soft palate, the posterior pharyngeal wall, and the lateral pharyngeal walls. All these structures show specific movements during speech and swallowing. Although the posterior and lateral pharyngeal walls are not affected by a cleft palate, the cleft palate disrupts the normal fusion of the velar muscles significantly affecting their movement during speech and swallowing. A vast number of scientific papers have focused on the diagnosis and management of cleft palate.

At the present time, it is universally accepted that patients with a cleft palate require the intervention of an interdisciplinary team. This special issue includes six papers addressing diverse aspects of the diagnosis and management of cleft palate.

One of the papers included in this special issue describes and discusses current controversies in the diagnosis and management of cleft palate and velopharyngeal insufficiency. Healthcare professionals from Chile, Mexico, and USA who work on cleft palate teams participated in this review. Imaging diagnosis and its importance for surgical planning and correction of velopharyngeal insufficiency, speech, and language pathology intervention for articulation disorders related with cleft palate and genetic aspects are reviewed in this paper.

The outcome of primary palatal surgery in children with cleft palate is addressed in another study from researchers from Korea. The differences between a syndromic and a nonsyndromic cleft palate are described. This study presents clinical outcomes of primary cleft palate surgery, including rate of oronasal fistula development, rate of velopharyngeal insufficiency, and speech outcomes. The results of this paper suggested that several factors, including cleft type, should be identified and comprehensively considered in order to establish an optimal treatment regimen for patients with cleft palate.

Imaging diagnosis has become an indispensable tool for assessing the velopharyngeal sphincter during speech. In a study from a group of researchers from Japan, the differences in velopharyngeal structure during speech as revealed by 3-Tesla magnetic resonance imaging movie mode are analyzed. This study describes the use of MRI movie as a powerful and valuable method which can be useful for studying velopharyngeal structure and function.

As technology keeps advancing, it is predictable that new imaging procedures will be readily available for routine clinical diagnosis in patients with cleft palate.

A group of researchers from Brazil submitted a paper studying the outcome of infants with Pierre Robin sequence managed with nasopharyngeal intubation. The results demonstrate that children managed with the protocol described in this study improved respiratory and feeding difficulties and presented low morbidity and no mortality during the first year of life.

Another study by a single author from Peru addresses one of the most severe complications of palatal surgical repair: a flap necrosis. Different factors which can play a role in flap failure are studied, including anatomical variations, section of the pedicle, tension, vascular thrombosis, surgical technique, infection, and malnutrition. The management of fistulas as a consequence of necrosis is also analyzed including different surgical procedures.

The dimension of the velopharyngeal space following surgical maxillary advancement is studied in a paper from a group of researchers from Turkey. In this study, Le Fort I osteotomy is compared with Zisser segmental osteotomy. Cephalometric analysis was used for assessing the velopharyngeal space. The results suggest that Zisser segmental osteotomy seems to be a reliable procedure for maxillary advancement with the advantage of not significantly increasing velopharyngeal space.

In sum, this group of papers provides useful clinical information for healthcare professionals interested in the interdisciplinary diagnosis and management of patients with cleft palate.

*Pablo Antonio Ysunza* *Maria Carmen Pamplona* *Gabriela Repetto*

